# The split protein phosphatase system

**DOI:** 10.1042/BCJ20170726

**Published:** 2018-12-06

**Authors:** Anne Bertolotti

**Affiliations:** MRC Laboratory of Molecular Biology, Francis Crick Avenue, Cambridge CB2 0QH, U.K.

**Keywords:** biochemical techniques and resources, intracellular signaling, protein phosphatases

## Abstract

Reversible phosphorylation of proteins is a post-translational modification that regulates all aspect of life through the antagonistic action of kinases and phosphatases. Protein kinases are well characterized, but protein phosphatases have been relatively neglected. Protein phosphatase 1 (PP1) catalyzes the dephosphorylation of a major fraction of phospho-serines and phospho-threonines in cells and thereby controls a broad range of cellular processes. In this review, I will discuss how phosphatases were discovered, how the view that they were unselective emerged and how recent findings have revealed their exquisite selectivity. Unlike kinases, PP1 phosphatases are obligatory heteromers composed of a catalytic subunit bound to one (or two) non-catalytic subunit(s). Based on an in-depth study of two holophosphatases, I propose the following: selective dephosphorylation depends on the assembly of two components, the catalytic subunit and the non-catalytic subunit, which serves as a high-affinity substrate receptor. Because functional complementation of the two modules is required to produce a selective holophosphatase, one can consider that they are split enzymes. The non-catalytic subunit was often referred to as a regulatory subunit, but it is, in fact, an essential component of the holoenzyme. In this model, a phosphatase and its array of mostly orphan substrate receptors constitute the split protein phosphatase system. The set of potentially generalizable principles outlined in this review may facilitate the study of these poorly understood enzymes and the identification of their physiological substrates.

## Introduction

Life depends on the controlled regulation of the activity of thousands of proteins. Protein phosphorylation is a post-translational modification that controls the fate, location and activity of the majority of cellular proteins. Protein phosphorylation occurs predominantly on serines and threonines, with kinases catalyzing the addition of a phosphate group and phosphatases reversing this. One can think of protein phosphorylation as a switch to turn signaling on or off through the antagonistic action of kinases and phosphatases. However, the reality may be more nuanced. It is likely that protein phosphorylation provides a versatile way to control protein function and fate through the constant antagonistic actions of kinases and phosphatases, both being most probably highly regulated. In that sense, the state of a given protein would oscillate between a phosphorylated and a non-phosphorylated state to adjust cellular functions to various signals resulting from changes in conditions.

There are ∼500 kinases in humans [1] and ∼189 phosphatases [2]. Unlike kinases which share a common catalytic fold and mechanism [1], phosphatases exhibit greater diversity of structures and catalytic mechanisms [2]. In contrast to kinases, which consist of a single polypeptide chain, phosphatases are found in complex with one or two non-catalytic subunits.

Protein phosphatase 1 (PP1) is an abundant protein that catalyzes most serine–threonine dephosphorylation in cells. Historically, PP1 has been purified following a procedure that dissociated it from its interactors [3]. The resulting enzyme is active against a variety of substrates leading to the erroneous notion that phosphatases are not selective. Because PP1 controls a large number of signaling events, it is hard to imagine why evolution would have designed a nonselective enzyme that controls so many aspects of life. I will argue that while PP1 is ubiquitous, it is not promiscuous. In contrast to the *in vitro* situation, it is believed that there is no free PP1 in cells, which is instead associated with one or two among an array of diverse non-catalytic subunits [4]. These complexes are the physiological holophosphatases and I will discuss how this heteromeric design led to exquisite selectivity.

I will begin this review with a historical overview of the discovery of protein phosphatases and integrate historical findings with recent observations.

## Discovery of protein phosphatase

Gerty Cori and Arda Green, working on glycogen phosphorylase, an enzyme that catalyzes the rate-limiting step of glycogenolysis, discovered protein phosphorylation by discovering the first phosphatase activity [5]. Phosphorylase can be isolated from muscle in two forms: an active form, phosphorylase *a* and an inactive form, phosphorylase *b*. Phosphorylase *b* becomes active upon the addition of AMP, whereas *a* is active without it. Remarkably, Cori and Green figured out that *a* contains a non-protein prosthetic group, which they reported correctly was covalently attached, because it could not be dissociated easily. Its removal needed an enzyme, contained in muscles and other tissues, which they named PR: the prosthetic group-removing enzyme [5]. The prosthetic group was, in fact, a phosphate and PR was the phosphate-removing enzyme (the phosphatase PP1). This discovery was also the first report of an allosteric regulation of an enzyme, although the concept of allostery was only formally articulated later [6]. Now, 75 years later, phosphorylase *a* is still widely used as a model substrate to study protein phosphatases. Yet, as we know, protein phosphorylation reversibly regulates most cellular proteins and there are thousands of phosphorylated sites [7]. Thus, many phosphatase substrates remained to be characterized.

The discovery of the first protein kinase followed, as often in science, a rather tortuous path. Ed Krebs trained as a post-doc with Cori and Green, but he struggled to produce active phosphorylase in his own laboratory. He was not the only one to fail replicating the results of Cori and Green (see references within [8]). Ed Krebs and Eddy Fischer converted their frustrations and misfortune into a puzzle: what makes phosphorylase *a* active in Cori and Green's preparation? Therein lies a frequent pattern underlying important scientific discoveries: Krebs and Fischer did not give up in the face of failure, they worked to understand what causes their problem.

Scrutinizing the differences between procedures, with rigor and diligence, Krebs and Fischer realized that the *a* form of phosphorylase appeared following filtration through filter paper. If this step was omitted, phosphorylase *b* was recovered. The effect of filtration was accounted for by the presence of metals in the paper: washing the paper abolished the effects, while adding paper ashes recapitulated the effect, indicating that the factor, required to activate phosphorylase, was inorganic. This led Krebs and Fischer to discover that they could convert their phosphorylase *b* into *a* upon incubation in a cell-free extract in the presence of a divalent metal ion and ATP [8]. Krebs and Fischer, with persistence and perseverance, converted their failures into a Nobel prize-winning discovery of broad significance.

The discovery of the phosphorylation of phosphorylase, which was the opposite reaction to the dephosphorylation reaction discovered by Cory and Green was, as we now know, the first illustration of a general principle that controls all aspects of life: the reversible phosphorylation of proteins.

## Discovery of PP1 and inhibitors

It is important to emphasize that seminal discoveries in the phosphatase field were made before DNA cloning became available. For many years, laboratories were measuring activities of different preparations, rendering direct comparisons difficult. The literature from this period is somewhat cryptic and confusing, but two excellent review articles summarize the paths that led to the discovery of serine/threonine phosphatases [9,10].

### Protein phosphatase 1

Another breakthrough in the path of the PP1 phosphatase discovery came, as often in science, serendipitously. In 1974, Howard Brandt, Derck Killilea and Ernest Lee noted a marked (up to 43 fold) activation of the phosphorylase *a* phosphatase upon combined precipitation with ammonium sulfate and ethanol [3]. ‘The activation of the enzyme … is shown to occur with the concomitant conversion of the enzyme from multiple molecular weight forms to a single form of lower molecular weight (M.W. ∼30 000)’. In these early days, the proteins that were removed by this purification procedure were considered to be contaminants.

### PP1 inhibitors and more phosphatases

The finding that partially purified phosphorylase phosphatase is inactivated by protein kinase A (PKA) (then known as ‘cyclic AMP-dependent protein kinase’) paved the way for the discovery of PP1 inhibitors [11]. Inactivation of partially purified phosphorylase phosphatase was found to be indirect, because phosphorylation of the enzyme could not be demonstrated [11]. Rather, the activity of phosphorylase *a* was inhibited by the phosphorylation of a potent and heat-stable inhibitor [11]. The following year, the purification of two phosphorylase *a* phosphatase inhibitors, Inhibitor-1 and -2, which were heat-stable and trypsin-sensitive, was reported [12]. Because phosphorylated Inhibitor-1 did not inhibit dephosphorylation of glycogen synthase or histones, and because Inhibitor-2 in the presence of Mn^2+^ inhibited glycogen synthase phosphatase activity, it was proposed that these inhibitors could confer substrate specificity [11].

In 1977, Philip Cohen and coworkers identified three phosphatases of different apparent molecular mass and substrate specificities that they named phosphatase 1, 2 and 3 [13]. They also showed that one of them, PP1, catalyzes dephosphorylation of many substrates involved in glycogen metabolism and proposed that, in this way, co-ordinated dephosphorylation events performed by the same phosphatase could inhibit glycogenolysis and stimulate glycogen synthesis [13]. Protein phosphatase 2 (PP2) had little activity on these substrates [13].

These findings shaped the future of phosphatase research by introducing the notion that phosphatases have broad substrate selectivity. Consolidating this idea, Thomas Ingebritsen and Cohen tested the selectivity of the four known serine/threonine phosphatases on many more substrates and concluded that ‘protein phosphatase-1 (PP1) and protein phosphatase 2A have very broad substrate specificities’ [14]. The differential sensitivities of phosphatases to the inhibitors were a distinctive property: PP1 was inhibited by low concentrations of Inhibitor-1 and Inhibitor-2, in contrast with PP2, which was unaffected by these proteins [15]. PP2 activity later became PP2A, PP2B and PP2C [16]. The broad substrate selectivity of the purified phosphatases led to the view that phosphatases were promiscuous.

### DARPP-32, another PP1 inhibitor

DARPP-32 is a phospho-protein, originally purified from rat brain with biochemical properties similar to inhibitor-1 [17]. Like inhibitor-1, DARPP-32 is soluble at acidic pH, heat-stable, has an acidic isoelectric point, a low content in hydrophobic amino acids, a low molecular mass and is natively unstructured [17]. These analogies led to the suspicion that DARPP-32 could also inhibit PP1. Indeed, it was found that DARPP-32 is as potent as Inhibitor-1 in inhibiting the dephosphorylation of phosphorylase *a* by PP1 [18].

## PP1 targeting subunits

Before it was realized that protein phosphorylation controls every aspect of life, the studies of phosphatases originated from work on glycogen metabolism. In contrast with PP2A and PP2C, which can be readily extracted, nearly all active PP1 in muscle is bound to particles of glycogen, myofibrils and the sarcoplasmic reticulum [19]. The glycogen-associated form of PP1 is a heterodimer composed of the PP1 catalytic subunit, which I will now refer to as PP1c, bound to a 161-kDa glycogen-binding (G, also called G_M_, because it was purified from muscle) subunit [19]. Knowing that the activities of key enzymes of glycogen metabolism (phosphorylase *a*, glycogen synthase b_1_ and b_2_, phosphorylase kinase α and β subunits) are controlled by reversible phosphorylation, the following notion emerged: the glycogen-binding subunit targets PP1c to glycogen to increase the concentration of these otherwise nonselective enzymes near the phospho-protein substrates, which are the enzymes regulating glycogen metabolism [19]. This concept is analogous to that of the signal peptide notion, which targets proteins to the endoplasmic reticulum [20]. In this way, the glycogen-regulatory subunit is like a postcode that sends PP1c to a location where dephosphorylation is needed. Since PP1c is capable of dephosphorylating many substrates, targeting PP1c to different subcellular locations could provide a mechanism to increase its concentration near its substrates and thereby confer a degree of specificity.

**Figure 1.**
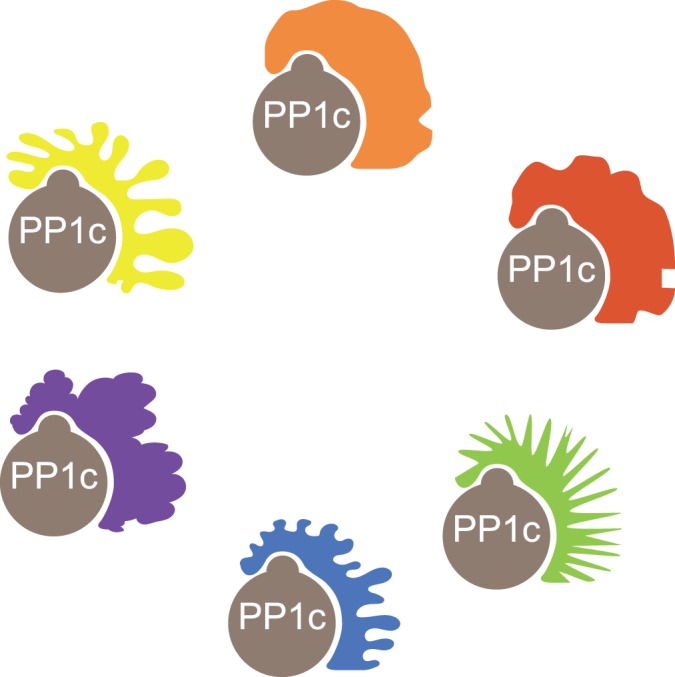
PP1 phosphatases are obligatory heteromers composed of shared catalytic subunits PP1c (gray) bound to one (or two) of many diverse non-catalytic subunits (colored). It is unclear how many PP1 holoenzymes exist, but it is estimated that there may be several hundred.

The concept of targeting subunits was extended with the purification of the smooth muscle myosin-bound phosphatase which contains two subunits in addition to PP1c [21]. Over the years, the range of PP1 functions has continued to expand well beyond glycogen metabolism and muscle contraction with gradual recognition that PP1 controls nearly all aspects of life [22]. More regulatory subunits of PP1 were identified: 15 different PP1 regulatory subunits had been identified by 1997 and regulatory subunits were proposed to target the catalytic subunit to particular regions of the cell, for example glycogen particles, myosin fibers or the nucleus [23]. A more recent search for PP1c interactors suggests that there may be several hundreds of regulatory subunits [24] leading to the notion that PP1 consists off a repertoire of hundreds of heteromeric enzymes composed of a shared catalytic subunit bound to one (or two) different regulatory subunits [25] (Figure 1). These regulatory subunits have not been well studied and identifying their functions is an open playground for future studies.

## PP1c catalysis

The three-dimensional structure of PP1c was solved in 1995, revealing a new fold and the catalytic mechanism [26,27]. PP1c is a metalloenzyme with two metal ions, Mn^2+^ and Fe^2+^, located at the base of a shallow cleft that can accommodate phospho-serine and phospho-threonine [26,27]. The active site is located at the bifurcation point of a Y-shaped groove, with one arm being hydrophobic and the other acidic. The PP1c inhibitor microcystin binds to three regions around the active site: the metal-binding site, the hydrophobic groove and the edge of the C-terminal groove, thus sterically blocking the active site [26], through a covalent interaction with cysteine [28]. The binding of microcystin, okadaic acid and Inhibitor-1 and -2 is mutually exclusive, indicating that they compete for the same binding site [27].

In contrast with the tyrosine phosphatases, as well as acid and alkaline phosphatases, PP1c dephosphorylation is catalyzed in a single step by a metal-activated water molecule. Metal ions stabilize negative charges and render the phosphate ester more susceptible to nucleophilic attack. It was noted that the surface of PP1c does not have a peptide-binding cleft, unlike serine/threonine kinases which possess a pronounced peptide-binding cleft important for substrate selectivity [27]. Thus, while the catalytic mechanisms of substrate dephosphorylation by PP1c have been elucidated, the molecular basis of substrate recognition remains largely unknown. It is also unclear if the non-catalytic subunits contribute to the catalytic mechanism.

## The functions of PP1 regulatory subunits

Various functions of regulatory subunits have been proposed, as reviewed below.

### Regulatory subunits as inhibitors

**Figure 2.**
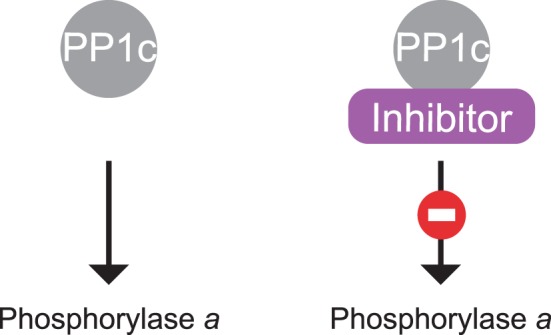
Regulatory subunits as inhibitors of PP1c. PP1c alone dephosphorylates phosphorylase *a*. When bound to an inhibitor, it fails to do so.

Inhibitor-1 was the first regulator of PP1. As described above, biochemical removal of Inhibitor-1 enhances PP1 activity towards phosphorylase *a* [3]. Subsequently, Inhibitor-2 [11], DARPP-32 [17] and Nuclear Inhibitor of Phosphatase (NIPP1) [29] were discovered. *In vitro*, they exhibit the same function as Inhibitor-1: they bind PP1c and inhibit the dephosphorylation of phosphorylase *a* by PP1. More recently, over 45 different proteins have been reported to bind to PP1c and to inhibit dephosphorylation of phosphorylase *a* [24]. Thus, the most commonly reported function of PP1 regulatory subunits is their inhibitory effect on the dephosphorylation of phosphorylase *a in vitro* (Figure 2). However, it is unlikely that so many different proteins have evolved to inhibit phosphorylase *a.* While inhibition of phosphorylase *a* dephosphorylation is a useful assay that led to the identification of many non-catalytic subunits of PP1, the physiological relevance of this activity has not been examined. This is an open question for future research.

### Regulatory subunits to target PP1c to subcellular location

**Figure 3.**
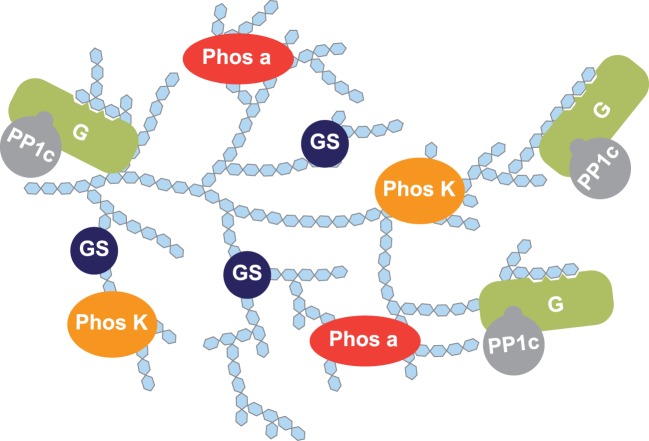
Targeting PP1c to glycogen. The G subunit (green) binds to both glycogen (light blue polymer) and PP1c, thereby targeting PP1c to glycogen. Phosphorylase *a* (Phos a, red), glycogen synthase (GS, navy blue) and phosphorylase kinase (Phos K, orange) are PP1c substrates. How PP1 recognizes these substrates is unknown.

As described in paragraph 4, the discovery of the glycogen-binding (G) subunit [19] lead to the proposition that targeting PP1c to glycogen, by direct binding of the glycogen-binding subunit to glycogen, increases PP1c concentration near the enzymes involved in glycogen metabolism (Figure 3). Following this example, many other PP1 regulatory subunits were identified and proposed to target PP1c to specific subcellular locations. In this model, selectivity increases as a result of an increase in the local concentration of PP1c.

As discussed above, many enzymes of glycogen metabolism (phosphorylase *a*, glycogen synthase b_1_ and b_2_, phosphorylase kinase α and β subunits) are regulated by PP1. Glycogen is a polymer of glucose and glycogen synthesis is tightly regulated to maintain glucose homeostasis. Thus, alterations of glycogen metabolism can have detrimental consequences on glucose metabolism, for instance diabetes. Therefore, one must wonder if targeting PP1c to glycogen is sufficient for the tight and highly regulated control of the dephosphorylation of the diverse enzymes in glycogen metabolism (Figure 3). I would like to suggest that in addition to targeting PP1c to glycogen, regulatory subunits may also target PP1c directly and selectively to each enzyme of glycogen metabolism and thereby provide the necessary molecular precision required to control physiology. At this point, the targeting function of the glycogen subunits to glycogen is well understood but glycogen is not a substrate of PP1c. How PP1c recognizes phosphorylase *a*, its historical substrate, remains unknown.

### Regulatory subunits as modifiers of substrate specificity

**Figure 4.**
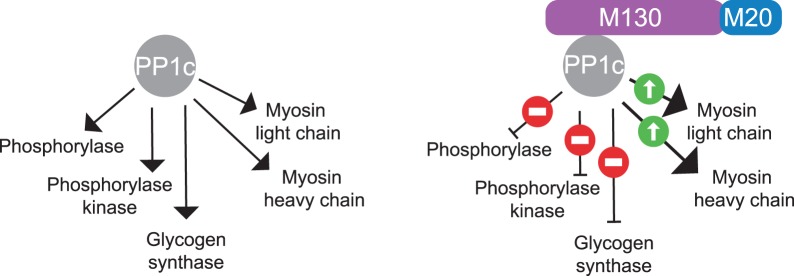
Summary of how the smooth muscle myosin regulatory subunits M130 and M20 change the substrate specificity of PP1c. *In vitro*, PP1c dephosphorylates a broad range of substrates: phosphorylase *a*, phosphorylase kinase, glycogen synthase. When PP1c is bound to M130 and M20, dephosphorylation of phosphorylase *a*, phosphorylase kinase, glycogen synthase is suppressed while dephosphorylation of the myosin light and heavy chains is enhanced.

In 1992, Dario Alessi and Cohen established a paradigm for the study of PP1 regulatory subunits [21]. They purified and characterized the major myosin phosphatase associated with the myofibrils of avian smooth muscle. They found that it is composed of PP1c complexed to two regulatory subunits, of 130 and 20 kDa, which are distinct from the G subunit [21]. The regulatory complex composed of the 130-kDa and 20-kDa subunits changes the substrate selectivity of PP1c by enhancing its activity towards myosin and suppressing its activity towards phosphorylase *a*, phosphorylase kinase and glycogen synthase [21]. These findings are summarized in Figure 4.

**Figure 5.**
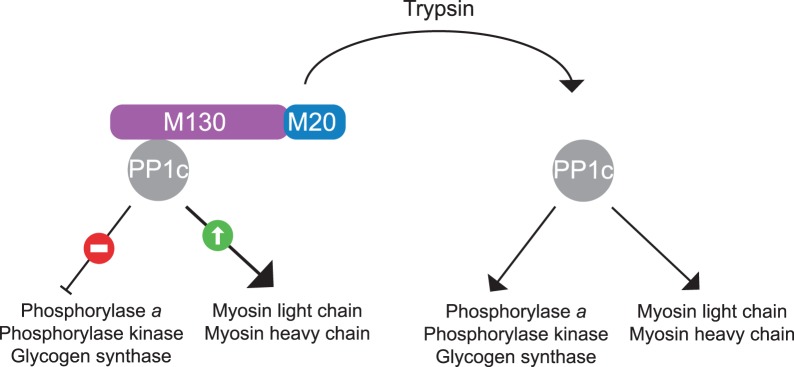
Trypsin converts myosin phosphatase into PP1c. Low concentrations of trypsin digest M130-M20, but spare PP1c. This assay has been used to remove non-catalytic subunits of PP1c, thereby generating an enzyme of broad substrate selectivity.

Interestingly, Alessi and colleagues exposed the trimeric myosin phosphatase to limited proteolysis, a treatment known to digest Inhibitor-1 [12] and the glycogen-targeting subunits [30], and tested the properties of the resulting products. They found that trypsin-proteolyzed myosin phosphatase had properties similar to PP1c: trypsin increased phosphorylase phosphatase activity and decreased myosin phosphatase activity. From this, they concluded that substrate-modifying activity was associated with trypsin-sensitive regulatory subunits [21] (Figure 5). The experimental paradigm developed by Alessi and Cohen consisting of purifying PP1c upon removal of its interactor by limited proteolysis has been followed to characterize other regulatory subunits [31]. While the work on myosin phosphatase proposes a function of regulatory subunits as modifiers of substrate selectivity, it is fascinating to note that more than 25 years have passed since this publication and the molecular basis of these findings have still not been elucidated.

### Regulatory subunits as scaffolds

Nuclei from bovine thymus contain a high level of PP1c in an insoluble fraction bound to two heat- and acid-stable proteins that inhibited the phosphorylase phosphatase activity of PP1c *in vitro* and therefore were named NIPP-1a and NIPP-1b [32]. These proteins are also called scaffolds [32].

### Regulatory subunits as biogenesis factors

Alessi and colleagues proposed in 1993 that Inhibitor-2 functions like a chaperone that helps the recombinant PP1c reach native-like activity [33]. Following this as a paradigm, SDS22 has also been proposed to function as a chaperone/biogenesis factor for PP1c [34].

## The PPP family

PP1c is a member of a family of related catalytic subunits (PPP) [16]. PP2A, like PP1, is a catalytic subunit of oligomeric enzymes [16,25]. They have a common heterodimeric core composed of a catalytic subunit, PP2A, and a scaffold [35]. The core binds to one of 26 B subunits, with the three subunits forming a specific holoenzyme [35]. PP2A phosphatases play important roles in cell cycle, cell proliferation and cancer [35]. Calcineurin is a calcium-regulated phosphatase that is involved in immune response. The catalytic subunit PP2B (also known as calcineurin A) assembles with a regulatory subunit (calcineurin B) [16]. Calcineurin is the target of the immunosuppressants cyclosporin A and FK506 [36]. These drugs do not directly bind to the phosphatase. The drugs bind to other proteins, cyclophilins. It is the drug–cyclophilin complex that inhibits calcineurin [36] by interfering with substrate recognition by binding to a surface important for substrate docking [37]. PP4 and PP6 are also catalytic subunits of heteromeric enzymes. In contrast, PP5 acts as a single polypeptide [16].

PPPs arose from a common ancestor [2] and share a catalytic mechanism whereby six highly conserved amino acids in the active site bind to two metal ions which are thought to activate a water molecule to initiate a nucleophilic attack on the phosphate [26,27]. With the exception of PP5, all PPPs form holoenzymes by binding to one or two non-catalytic subunits. Many non-catalytic subunits exist, creating a large number of phosphatases. While the catalytic domain is conserved, each enzyme associates with a distinct set of regulatory subunits. The physiological substrates of the hundreds of holophosphatases remain to be identified.

Yeast has one PP1c, Glc7, mammals have three. They are referred to as PP1 α, β and γ isoforms but should, in fact, be called paralogs as they are encoded by three different genes localized on chromosome 11, 2 and 12, respectively (https://www.genenames.org/cgi-bin/genefamilies/set/693). These paralogs arose recently [2] and their sequences are very similar, with some divergences localized mostly in the amino- and carboxy-terminal regions. The divergent regions in the different PP1 paralogs could contribute to select different sets of interactors [38]. PP2A has two paralogs, α and β, whereas PP2B has three, α, β and γ. These PPP paralogs are highly related, as they also arose recently [2].

## A case study: the regulatory subunits of eIF2α phosphatases

### Discovery of eIF2α phosphatases

Phosphorylation of the α subunit of eukaryotic translation initiation factor 2 (eIF2α) is a vital first line of defense against many stresses. In mammals, four different eIF2α kinases sense various stresses and respond by phosphorylating eIF2α to reduce protein synthesis [39]. GCN2, the only eIF2α kinase present in yeast, is induced by amino acid shortage [39]. Mammals have three additional eIF2α kinases to protect against a broader range of insults: PKR is activated upon viral infection, HRI by heme deficiency, and PEK/PERK in response to protein misfolding stress in the endoplasmic reticulum [40,41]. Phosphorylated eIF2α sequesters eIF2B, preventing the exchange of eIF2-GDP to eIF2-GTP, an essential step in the recycling of eIF2 [39]. Thus, phosphorylation of eIF2α reduces translation initiation, a rapid and vital response to many stresses. In mammals, two heterodimeric eIF2α holophosphatases antagonize the action of the four eIF2α kinases: each is composed of a non-catalytic subunit, the stress-inducible PPP1R15A (R15A) [42,43] or the constitutive PPP1R15B (R15B) [44], bound to the catalytic subunit protein phosphatase 1 (PP1c).

Knowledge of the function of the mammalian eIF2α phosphatases stems from their homology to a viral protein. In 1994, Joany Chou and Bernard Roizman discovered the function of the γ_1_34.5 gene of herpes simplex virus: mutants lacking a functional γ_1_34.5 gene failed to escape the shutdown of protein synthesis that followed viral infection [45]. They noted that the carboxy-terminal domain of γ_1_34.5 is homologous to the carboxy-terminal domain of the product of the growth arrest and DNA damage gene Gadd34 (now PPP1R15A) [45] and indeed later showed that the carboxy-terminal domain of Gadd34 can substitute for the homologous domain of γ_1_34.5 [46]. Chou and colleagues found that shutdown of protein synthesis following viral infection was mediated by PKR which phosphorylated eIF2α [47]. The Roizman laboratory then performed an unbiased yeast two-hybrid screen and found that Gadd34 and γ_1_34.5 interacted with PP1c [48]. The functional relevance of this interaction was supported by the finding that okadaic acid inhibited protein synthesis, indicating that a phosphatase was involved in the process (directly or indirectly). Moreover, lysates of cells infected with wild-type virus, but not a mutant lacking a functional γ_1_34.5 protein, had higher eIF2α phosphatase activity. This activity was sensitive to Inhibitor-2 [48]. Conversely, γ_1_34.5-containing lysates had a lower phosphatase activity towards phosphorylase *a* [48]. In subsequent work, the same authors identified a canonical PP1c-binding peptide containing a KVRF motif and concluded that γ_1_34.5 has the attributes of a PP1 regulatory subunit [49].

Shirish Shenolikar and colleagues later recapitulated key findings from the Roizman laboratory and showed that PPP1R15A formed a complex with PP1c [43]. David Ron and colleagues also provided evidence, using cellular assays, that PPP1R15A dephosphorylates eIF2α following stress in the endoplasmic reticulum [42], similar to what Roizman had described for γ_1_34.5 following viral infection [45]. The discovery of a paralogue, sharing ∼23% sequence identity with PPP1R15A, and known as PPP1R15B (CReP), followed [44]. Unlike PPP1R15A which is inducible by a variety of stresses, PPP1R15B is constitutively expressed [44]. Cells lacking functional PPP1R15A [50] or PPP1R15B [51] have increased levels of eIF2α phosphorylation.

### Structures of PP1c bound to fragments of non-catalytic subunits

As described above, there is abundant evidence that in cells, the non-catalytic subunits of PP1c, PPP1R15A and PPP1R15B, similar to γ_1_34.5, enhance dephosphorylation of eIF2α. However, how these proteins achieve their function was unknown until recently.

Following seminal work from David Barford's laboratory, who solved the structure of a fragment of the G-regulatory subunit bound to PP1c and thereby discovered the RVXF conserved in many regulatory subunits [52], structures of the RVXF region of other regulatory subunits, including those of PPP1R15s bound to PP1c, were determined [38,53–59]. They confirmed how regulatory subunits bound to PP1c, but they do not shed light on the functions of the holoenzymes because only small fragments of regulatory subunits, often less than 20 amino acids in length, were seen [38,53–59]. Some studies of PP1c complexes bound to small fragments of regulatory subunits incorrectly referred to such complexes as holoenzymes. There is currently no published structure of a PP1 holoenzyme.

The structure of Inhibitor-2 bound to PP1c explains its function: Inhibitor-2 binding occluded the active site of PP1c, thereby revealing how it inhibited PP1c: substrates cannot reach the active site when Inhibitor-2 was bound to PP1c [53]. Importantly, Inhibitor-2 was structured when bound to PP1c, with two helices lying across the substrate-binding cleft [53].

Keeping in mind the variety of functions attributed to regulatory subunits (inhibitors, targeting to subcellular locations, modifiers of substrate selectivity, scaffold or biogenesis factors), how regulatory PPP1R15A and PPP1R15B could affect eIF2α phosphorylation in cells was unclear until recently.

### Functional characterization of non-catalytic subunits of eIF2α phosphatases

Shenolikar and colleagues demonstrated that recombinant PPP1R15A inhibited dephosphorylation of phosphorylase *a* by PP1c [43]. A similar inhibitory activity was observed by Roizman in cells with γ_1_34.5, who also showed that γ_1_34.5 enhanced dephosphorylation of eIF2α [48]. However, such an activity was not observed with purified recombinant PPP1R15A *in vitro*: PPP1R15A had no effect on eIF2α dephosphorylation when added to PP1c. At this point, only one function of PPP1R15A, its inhibitory activity, was replicated *in vitro* [43].

**Figure 6.**
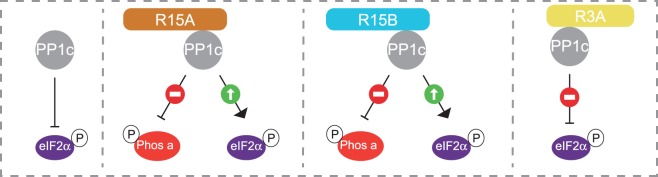
An assay with recombinant proteins recapitulates the function and selectivity of PPP1R15–PP1c holophosphatases. PPP1R15A: R15A. PPP1R15B: R15B. At physiological concentrations, PP1c does not dephosphorylate eIF2α. However, R15A–PP1c and R15B–PP1c are active and selective eIF2α phosphatases: they dephosphorylate eIF2α but not phosphorylase *a*. An unrelated holoenzyme, R3A-PP1c, does not dephosphorylate eIF2α. Thus, the function and selectivity of these holoenzymes can be recapitulated *in vitro* with purified proteins.

With a large body of cellular data from various laboratories indicating that PPP1R15A and PPP1R15B enhance eIF2α dephosphorylation, it became important to reconstitute this function with recombinant proteins. We first replicated the findings of Shenolikar, finding that recombinant PPP1R15A inhibited dephosphorylation of phosphorylase *a* by PP1c [60]. We also found that PPP1R15B had a similar inhibitory activity towards phosphorylase *a*. We then aimed to develop an assay that revealed the function of these regulatory subunits towards eIF2α. We found that, when using sub-stoichiometric concentration of PP1c, the dephosphorylation of eIF2α depends on the addition of PPP1R15A or PPP1R15B (Figure 6). Ron and colleagues reported that actin was required to confer selectivity to the holoenzyme [58], but the molecular basis for such a function remains unclear. In our assay, we observed that at high concentrations (stoichiometric amounts of PP1c to substrates), PP1c alone dephosphorylates eIF2α and phosphorylase *a*. However, it is important to keep in mind that it has been known since 1974 that PP1c is not found in isolation in cells, but that it exists in protein complexes [3]. Therefore, purified PP1c is not likely to have the same properties as native holoenzymes. In contrast with isolated PP1c, the reconstituted holophosphatases, PPP1R15A–PP1c and PPP1R15B–PP1c, were highly selective: they were unable to dephosphorylate phosphorylase *a*. Conversely, an irrelevant holoenzyme composed of PPP1R3A (the G-targeting subunit) bound to PP1c was unable to dephosphorylate eIF2α [60]. The reconstituted holophosphatases exhibited both the function and selectivity of their cellular counterparts (Figure 6).

### Molecular basis for the selectivity of non-catalytic subunits of eIF2α phosphatases: high affinity for their substrate

**Figure 7.**
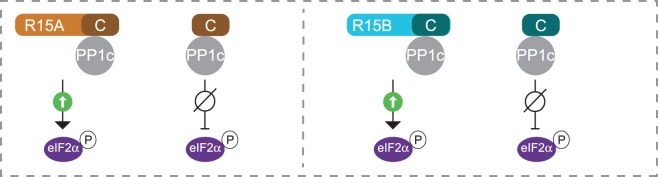
Complexes composed of carboxy-terminal fragments of PPP1R15s are capable of recruiting PP1c but are not functional. Holoenzymes assembled with large fragments of PPP1R15s (R15A or R15B) are functional. The carboxy-terminal regions of R15s bind PP1c, but the resulting complexes are not functional.

After having reconstituted the function and selectivity of eIF2α holophosphatases, we were in a position to decode the function of the non-catalytic subunits. In agreement with what had been seen before, we found that the carboxy-terminal region of PPP1R15s, which contains a canonical RVxF motif, binds to PP1c with high affinity [60]. However, whilst this region is necessary and sufficient to recruiting PP1c, we found that complexes containing only the carboxy-terminal region of PPP1R15s (PPP1R15A^513–636^ and PPP1R15B^636–698^) were not functional because they did not allow PP1c to dephosphorylate eIF2α [60]. This demonstrated that PPP1R15s must have other essential functions, in addition to the recruitment of PP1c (Figure 7).

We found that an essential function encoded by the amino-terminal region of PPP1R15s is to provide a high-affinity binding site for its substrate [60]. A study in yeast has suggested a binding site for eIF2α in the conserved carboxy-terminal region on PPP1R15A and its viral counterparts [61]. However, this binding was not observed with recombinant PPP1R15A [60]. In human cells, PPP1R15A also binds to eIF2α through its PEST repeats, as we observed for recombinant PPP1R15A [60].

**Figure 8.**
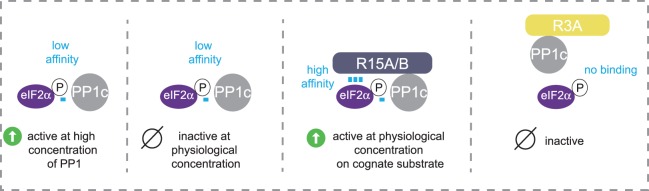
Understanding the selectivity of PP1 holophosphatases and the substrate specifier functions of non-catalytic subunits. PP1c has low affinity for phosphorylated eIF2α, explaining why at physiological concentrations it is unable to dephosphorylate this substrate. At high concentration, binding can occur, enabling dephosphorylation. PPP1R15s (R15A/B) provides a high-affinity receptor for eIF2α, enabling dephosphorylation at physiological concentrations of PP1c. The unrelated subunit R3A prevents PP1c binding to eIF2α and its dephosphorylation.

We found that while PP1c has only a low affinity for eIF2α, that of the PPP1R15–PP1c was much higher. Importantly, when PP1c was bound to an irrelevant non-catalytic subunit, such as PPP1R3A, it did not bind to eIF2α [60] (Figure 8). This provides the molecular basis for the selectivity of regulatory subunits and explains an old conundrum: how a regulatory subunit can function both as an inhibitor of PP1c towards a non-cognate substrate and increase dephosphorylation of the cognate substrate. The regulatory subunit provides high affinity to recruit the cognate substrate and to decrease the low affinity of PP1c towards non-cognate substrates. Keeping in mind that the bulk of PP1c is not free in cells [3], this shows that holophosphatases have been designed for exquisite selectivity, with the non-catalytic subunit being a high-affinity substrate receptor.

The molecular basis for the selectivity of PPP1R15–PP1c holoenzymes may also apply to other holoenzymes. In light of the findings obtained while studying the eIF2α phosphatases, I can propose the following interpretation of an earlier study on the myosin phosphatase [21] (Figure 4). Regulatory subunits M130 and M20 enhance PP1c activity towards myosin by providing a high-affinity receptor for myosin. Upon binding to PP1c, M130 and M20 decrease the low affinity of PP1c for non-cognate substrates (phosphorylase *a*, phosphorylase kinase and glycogen synthase), thereby inhibiting their dephosphorylation.

## Selective inhibitors of PP1 holophosphatases

PP1c is the catalytic subunit of hundreds of holophosphatase complexes. As a result, catalytic inhibitors of PP1c cannot be selective. Therefore, phosphatases have been traditionally overlooked by drug discovery programs, while kinases are among the top targets in pharmaceutical industry [62]. Because protein phosphorylation regulates virtually every signaling event in a cell, phosphatases could be attractive drug targets.

I work on protein quality control systems, which are the major cellular defense mechanisms against misfolded proteins [63]. Accumulation of proteins in abnormal conformations is the hallmark of most human neurodegenerative diseases including Alzheimer's, Parkinson's and Huntington's disease as well as amyotrophic lateral sclerosis (ALS). Boosting protein quality control mechanisms could be useful to reduce the load of misfolded proteins in these diseases [64]. Looking for an approach to do so, we found a small molecule, Guanabenz, that protected cells from otherwise lethal accumulation of misfolded proteins in the endoplasmic reticulum (ER stress) [65]. Guanabenz bound to and inhibited PPP1R15A. As a result, it prolonged phosphorylation of eIF2α and the duration of translation attenuation resulting from stress, giving cells more time and resources to recover.

Guanabenz, an α2-adrenergic agonist, was used to treat hypertension [66]. In a medicinal chemistry effort, we engineered out its adrenergic activity and developed Sephin1, a selective PPP1R15A inhibitor [67]. Like Guanabenz crossed the blood–brain barrier, was orally available and when given to mice, protected them from two conditions associated with ER stress, Charcot-Marie-Tooth 1B and ALS caused by a mutation in superoxide dismutase [67].

This showed that selective inhibition of a phosphatase can be achieved by targeting its non-catalytic subunit. We next thought that the same paradigm could be used to inhibit other phosphatases. However, as there were no methods available to enable the identification of selective inhibitors of non-catalytic subunits, there was no obvious path towards generalizing this concept.

This represented both a challenge and an opportunity. Realizing that we knew little about the function of PPP1R15s, we reasoned that if we wanted to develop assays to identify selective phosphatase inhibitors by targeting non-catalytic subunits, we needed to understand their function. This led us to reconstitute eIF2α holophosphatases with recombinant proteins and to elucidate their function. As discussed above, we found that PPP1R15 provided the substrate receptor function to the holoenzyme [60]. With knowledge of the function of PPP1R15s, we could begin to elucidate how Guanabenz and Sephin1 inhibit PPP1R15A. We found that they bound to an amino-terminal site of the protein, induced a conformational change, which impaired substrate recruitment [60]. This indicates that Guanabenz and Sephin1 are allosteric inhibitors of PPP1R15A.

**Figure 9.**
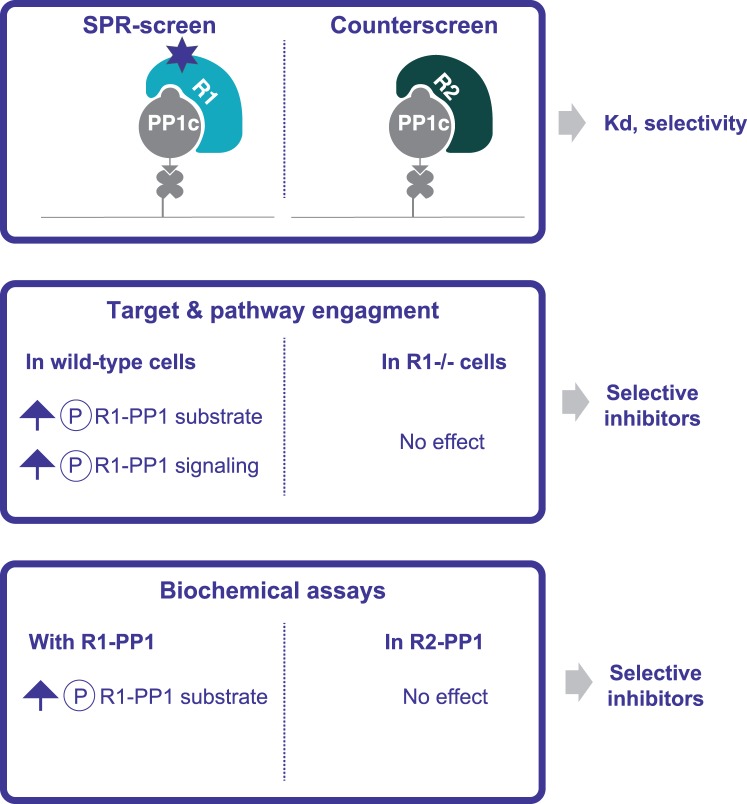
Assays to enable identification of selective phosphatase inhibitors. PPP1R1 (R1) and PPP1R2 (R2) are non-catalytic subunits. An SPR screen enables the identification of molecules binding selectively to a holophosphatase of interest, R1-PP1c, and a counter-screen with a different phosphatase, R2-PP1c, filters out nonselective binders. Cell-based assays consisting of monitoring increased phosphorylation of the substrate of the R1-PP1 phosphatase or downstream signaling events select for compounds capable of inhibiting their target in cells. The same assays performed in cells knockedout for R1 identifies on-target compounds. Biochemical dephosphorylation assays validate the mechanism of action and selectivity of the inhibitors.

Having been able to reconstitute selective and functional holoenzymes, we then developed an assay for target-based discovery of inhibitors of non-catalytic phosphatase subunits. We used surface plasmon resonance (SPR) to screen for selective serine/threonine phosphatase inhibitors. Different holoenzymes could be reconstituted on an SPR CHIP and used to screen for selective binders. The latter could then be tested in cell-based assays to measure target (accumulation of phospho-substrate) and pathway (downstream signaling events) engagement. The same experiments could be conducted with knockout cells to filters off-target molecules. The mechanisms of action of the inhibitors could then be validated in biochemical assays. This outlines a drug discovery platform for selective phosphatase inhibitors (Figure 9).

Our previous work highlighted the benefit of PPP1R15A inhibition. However, because PPPR15A expression is inducible, PPP1R15A inhibition is restricted to diseases where it is expressed. Therefore, we became interested in targeting constitutively expressed PPP1R15B, because we suspected that selective inhibition of PPP1R15B would circumvent the limitations associated with PPP1R15A inhibition, while conferring a proteostatic benefit. This idea was supported by earlier findings showing that knockdown of PPP1R15B protected cells against diverse stresses [44]. Although functionally related, PPP1R15A and PPP1RB share only 23% sequence identity. Thus, we could hope to isolate selective inhibitors of PPP1R15B. We identified Raphin1, a molecule that bound PPP1R15B–PP1c with 30-fold higher affinity than PPP1R15A–PP1c. In cells, Raphin1 inhibited PPP1R15B–PP1c and induced transient accumulation of phosphorylated eIF2α, resulting in transient attenuation of protein synthesis. The effects of Raphin1 were transient because eIF2α phosphorylation enabled translation of PPP1R15A, which, in a negative feedback, dephosphorylated eIF2α [68]. Inhibition of PPP1R15B mimicked the transient translation attenuation that resulted from ER stress, but in the absence of such a stress. Raphin1 crossed the blood–brain barrier and was orally available. In mice, it attenuated the misfolding of mutant huntingtin by increasing the cells' ability to fight this disease-causing protein. As a result, it reduced the disease phenotype [68].

This work establishes the power and benefit of inhibiting PPP1R15s. PPP1R15A inhibitors may be useful in diseases where it is expressed, such as those involving ER stress, while PPP1R15B inhibitors may be useful for disorders where the abnormally folded proteins accumulate outside the ER [69]. The assays and the platform developed to identify PPP1R15B inhibitors can, in principle, be used to all phosphatases. However, it is important to note that the substrates of most holophosphatases are unknown. Therefore, it will be important to gain knowledge about this class of enzymes.

## The split protein phosphatase system (SPS)

Reflecting on the path that led to the discovery of PPP1R15 inhibitors, it all started serendipitously. We did not set out to discover how to inhibit phosphatases. It is the discovery of PPP1R15A inhibitors that perked my interest in this class of enzymes. With the discovery of inhibitors, we gained tools to explore the biology. This led us to develop assays and a drug discovery platform. The druggability of these enzymes reinforces the need to characterize these enzymes, and their substrates and knowledge in this area will, in turn, open new avenues in cell biology as well as in drug discovery. I believe that we should not consider basic research as separate to drug discovery. As Louis Pasteur said « Il n'y a pas d'un côté la recherche fondamentale et de l'autre la recherche appliquée. Il y a la recherche et les applications de celle-ci, unies l'une à l'autre comme le fruit de l'arbre est uni à la branche qui l'a porté » (*There does not exist a category of science to which one can give the name applied science. There is science and the applications of science, bound together as the fruit of the tree which has borne it.)*.

**Figure 10.**
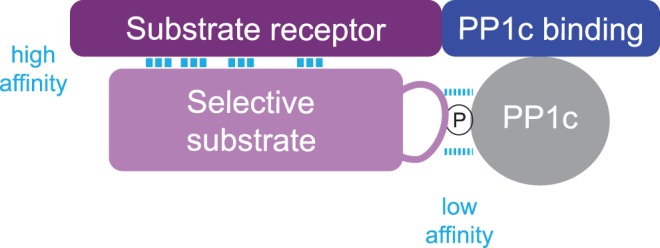
The split protein phosphatase system (SPS). PP1 holoenzymes are composed of a catalytic subunit PP1c bound to a non-catalytic subunit which is composed of at least two modules: a substrate receptor module and a PP1c-binding module. Additional modules can be added such as a glycogen-targeting module or other to target the complex to a specific subcellular location.

How can we use what we have learned so far? In light of the findings that provided the molecular basis for the function and selectivity of eIF2α phosphatases, a model emerges. The non-catalytic subunits of PP1 holoenzymes appear to be modular proteins: one domain binds PP1c and another serves as a high-affinity substrate receptor. Additional functions can be added to this basic design, such as targeting to a subcellular destination (glycogen for example). This model implies that PP1 phosphatases are split enzymes with both subunits being essential (Figure 10). Why did phosphatases evolve as heteromeric enzymes? One may speculate that assembling diverse substrate receptor units on a single catalytic unit minimizes the genetic information required to create functional diversity and opportunities for fine regulation.

## The SPS provides a unified model that reconciles diverse function of regulatory subunits

As discussed above, regulatory subunits were initially defined as inhibitors. This came about because PP1c was extensively purified, dissociated from the other components of physiological and selective holophosphatase complexes, and dephosphorylation reactions were performed on phosphorylase *a*.

**Figure 11.**
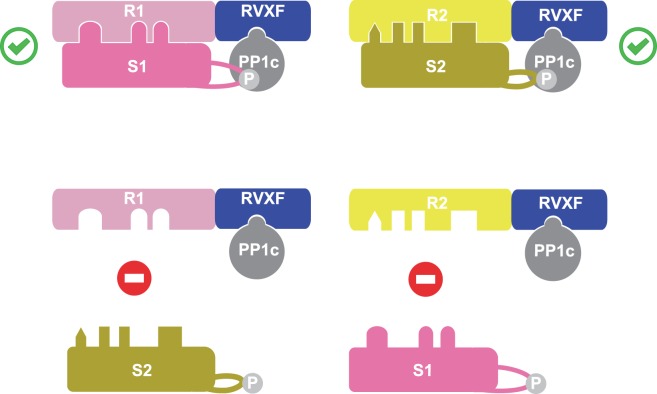
The split phosphatase system explains the dual function of non-catalytic subunits. R1 (PPP1R1) is the substrate receptor of the holophosphatase that recruits a specific substrate S1 and PP1c to enable selective dephosphorylation. Likewise, R2 (PPP1R2) is the substrate receptor for S2. R1 is inhibitory towards S2 and R2 is inhibitory towards S1.

As many studies have shown, many proteins containing a binding site for PP1c inhibit PP1c from dephosphorylating phosphorylase *a*. However, it is unlikely that hundreds of proteins have evolved to inhibit the dephosphorylation of phosphorylase. The split phosphatase model explains the dual functions of non-catalytic subunits of PP1: providing high-affinity for the cognate substrate and, consequently, inhibiting the low activity of the catalytic subunit on non-cognate substrate (Figure 11). It is a simple key-lock model governed by affinities: only the right key can open a given door. This model could apply to PP2A, PP2B, PP4 and PP6, since they are all split enzymes.

There may well be a few *bona fide* inhibitors of PP1c among the hundreds of non-catalytic subunits, which, like Inhibitor-2, may occlude the catalytic site. However, the vast majority of non-catalytic subunits are probably not inhibitors but orphan substrate receptors. This idea provides a simple framework for uncovering the functions of holophosphatases. The assays we have developed to elucidate the function and substrate selectivity of PPP1R15 are applicable to all phosphatases and should enable that rapid progress is this understudied area of biology.

## Abbreviations

ALS: amyotrophic lateral sclerosis
ER: endoplasmic reticulum
eIF2α: eukaryotic translation initiation factor 2
GS: glycogen synthase
PP1: protein phosphatase 1
PP2: protein phosphatase 2
PR: prosthetic group
SPR: surface plasmon resonance
SPS: Split Protein Phosphatase System
